# A Decade in Review after Idiopathic Scoliosis Was First Called a Complex Trait—A Tribute to the Late Dr. Yves Cotrel for His Support in Studies of Etiology of Scoliosis

**DOI:** 10.3390/genes12071033

**Published:** 2021-07-01

**Authors:** Nelson L. S. Tang, Matthew B. Dobbs, Christina A. Gurnett, Yong Qiu, T. P. Lam, Jack C. Y. Cheng, Nancy Hadley-Miller

**Affiliations:** 1 KIZ/CUHK Joint Laboratory of Bioresources and Molecular Research in Common Diseases, Department of Chemical Pathology, Li Ka Shing Institute of Health Sciences, The Chinese University of Hong Kong, Hong Kong SAR, China; 2 Functional Genomics and Biostatistical Computing Laboratory, CUHK Shenzhen Research Institute, Shenzhen 518000, China; 3 Dobbs Clubfoot Center, Paley Orthopedic and Spine Institute, West Palm Beach, FL 33401, USA; mdobbs@paleyinstitute.org; 4 Department of Neurology, Washington University in St Louis, St Louis, MO 63110, USA; gurnettc@wustl.edu; 5 Department of Spine Surgery, The Affiliated Drum Tower Hospital of Nanjing University Medical School, Nanjing 210000, China; scoliosis2002@sina.com; 6 Department of Orthopaedics & Traumatology and SH Ho Scoliosis Research Lab, Joint Scoliosis Research Center of the Chinese University of Hong Kong and Nanjing University, The Chinese University of Hong Kong, Hong Kong SAR, China; tplam@cuhk.edu.hk (T.P.L.); jackcheng@cuhk.edu.hk (J.C.Y.C.); 7 Department of Orthopedics, University of Colorado Anschutz Medical Campus, Aurora, CO 80012, USA; Nancy.Miller@ChildrensColorado.org

**Keywords:** idiopathic scoliosis, genetic predisposition, complex trait, model animal, genome wide association study, genetic linkage study

## Abstract

Adolescent Idiopathic Scoliosis (AIS) is a prevalent and important spine disorder in the pediatric age group. An increased family tendency was observed for a long time, but the underlying genetic mechanism was uncertain. In 1999, Dr. Yves Cotrel founded the Cotrel Foundation in the Institut de France, which supported collaboration of international researchers to work together to better understand the etiology of AIS. This new concept of AIS as a complex trait evolved in this setting among researchers who joined the annual Cotrel meetings. It is now over a decade since the first proposal of the complex trait genetic model for AIS. Here, we review in detail the vast information about the genetic and environmental factors in AIS pathogenesis gathered to date. More importantly, new insights into AIS etiology were brought to us through new research data under the perspective of a complex trait. Hopefully, future research directions may lead to better management of AIS, which has a tremendous impact on affected adolescents in terms of both physical growth and psychological development.

In 2005, Tang and Miller et al. received grant support from the Fondation Yves Cotrel in the Institut de France to investigate the genetic etiology of adolescent Idiopathic Scoliosis (AIS) [1]. During an academic tour to Hong Kong and China, we presented to Dr. Cotrel our new genetic concept for scoliosis etiology. Together with the breakthroughs of the time, such as the completion of Human Genome Project and International HapMap Project, Dr. Cotrel was convinced that it would be a “Prime Time” to forward genetic research related to AIS. The prevailing concept of AIS etiology at that time was that the disorder was due to one or two genes with major effects [2,3]. Conversely, we argued that AIS might be caused by an interplay of multiple genes and environmental factors. These ideas and hypotheses were later published in a paper titled “Genetic Association of Complex Traits, using Idiopathic Scoliosis as an Example” [4]. Although similar ideas were suggested, it was the first time that AIS was explicitly called a complex trait. Now, more than a decade has passed, and therefore, we took this opportunity to review how far our original hypothesis was supported and where we are in terms of the “Prime Time” of genetic research in AIS while we remember the contributions and teachings of Dr. Cotrel.

## 1. Setting the Scene

**Figure 1:**
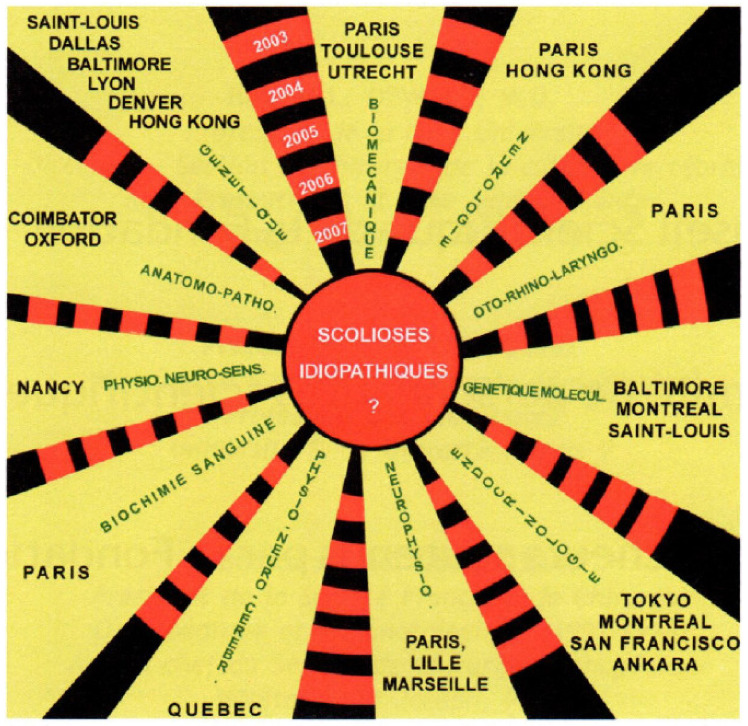
In 2007, Dr. Cotrel and team listed 10 potential categories of underlying causes of scoliosis. It was a key mission of the Cotrel Foundation to find out the primary etiology of adolescent idiopathic scoliosis (AIS). They include genetics, biomechanics, neurology, Oto-Rhino-laryngology, molecular biology, endocrinology, neurophysiology, biochemistry, sensory physiology, and anatomical pathology (courtesy of the Cotrel Foundation).

**Figure 2:**
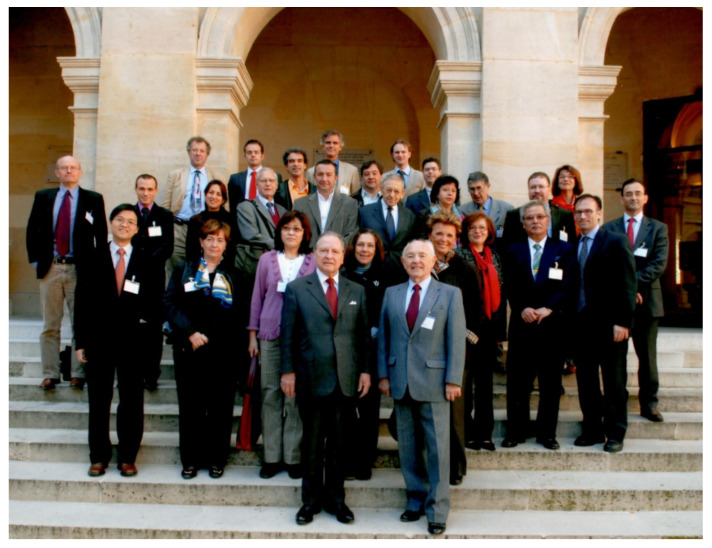
A group photo of international research colleagues and Dr. Cotrel (right on the first row) taken during 2007 Cotrel Foundation Annual Scientific meeting (courtesy of the Cotrel Foundation).

**Figure 3:**
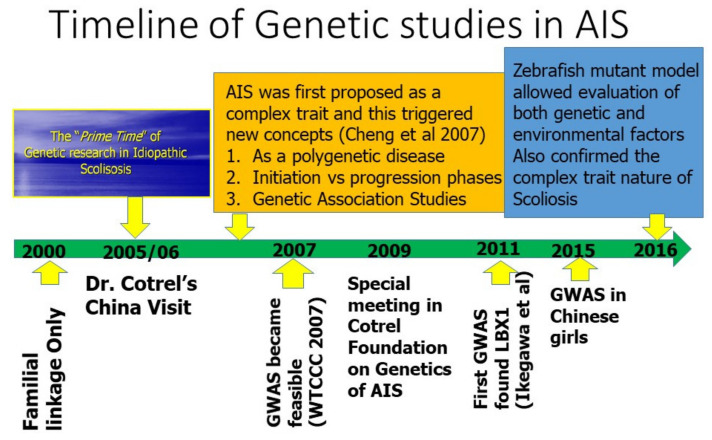
Timeline of genetic research milestones of AIS showing the essential role played by Cotrel Foundation in cultivating complex trait model of AIS.

Adolescent Idiopathic Scoliosis (AIS) is the most common form of spinal deformity [5]. It affects up to 4% of otherwise healthy adolescent girls in the population. However, there is still little understanding about the etiology of AIS. Many hypotheses were proposed which included genetic predisposition, growth, and hormonal disturbances, and developmental neuromuscular dysfunction (as illustrated in Figure 1 by Dr. Cotrel). Based on his categorization, The Cotrel Fondation gathered basic scientists and clinical researchers in Paris every year to discuss and propose primary etiologic mechanisms for AIS based on our area of expertise (as illustrated in Figure 2). These annual gatherings snowballed into a series of genetic studies into the etiology of AIS (as illustrated in Figure 3).

Although the tendency of AIS to run in families was described for a long time, the mechanism and mode of inheritance were unknown. Linkage analyses were carried out by different research groups to locate the causative genetic loci. Both parametric and nonparametric linkage analysis were applied to study familial forms of AIS and resulted in different modes of inheritance, including autosomal dominant and X-linked while multiple loci in chromosomes 9, 16, 17, 19, and X and others were implicated as the loci of major AIS disease genes [6,7,8,9]. Although some progress was made in understanding the candidate genetic loci, the familial aggregation pattern is not fully understood, and few highly confident causative genes were identified.

## 2. AIS Was First Considered as a Complex Trait

Genetic susceptibility for AIS is confusing and its inheritance mechanism is uncertain, however, it is now widely accepted that the complex trait (a polygenic inheritance working together with environmental factors) fits well with AIS. When we put forward this bold new theory in 2007 [4], we borrowed the concept from the case of breast cancer in which high penetrance genes (BRCA1 and BRCA2) were recognized as causing the autosomal dominantly inherited familial breast cancer. Excluding the familial cases, weak but significant genetic predisposition was established for the majority of sporadic breast cancer patients, as evident in the Claus model, and subsequently developed models [10,11]. With reference to these concepts, we hypothesized that genetic susceptibility for AIS potentially also operates in two forms: (a) rare familial scoliosis due to single gene defects, and (b) low penetrance genetic variants acting collectively accounting for genetic predisposition in the majority of “sporadic AIS”.

**Figure 4:**
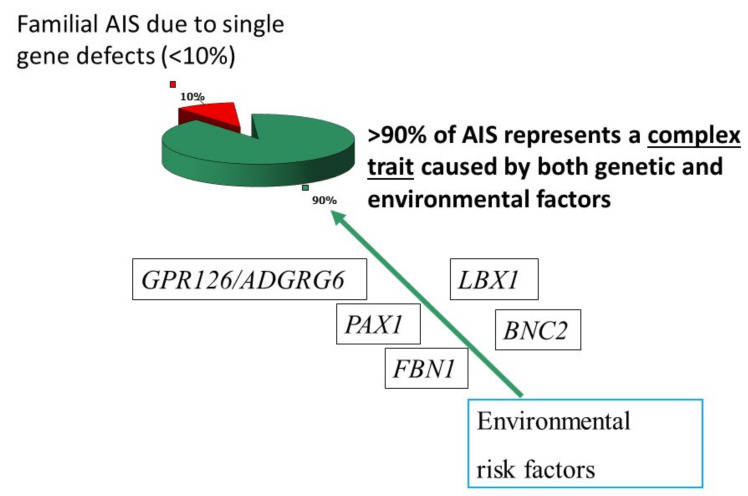
Genetic predisposition for AIS under perspective of a complex trait model. Plausible genes contributing to scoliosis are also shown of which many were identified by GWAS.

Figure 4 is an updated complex trait model that we put forward in the 2007 paper [4] after incorporating information from the latest research efforts in AIS. A number of AIS predisposition genes were discovered during this decade, and they are represented in the model. Implications of individual genes will be further discussed in this review article.

## 3. A High Heritability of Liability to AIS

Unlike other common diseases, there was a concern that a specific value of heritability was not yet determined for AIS [4,12,13]. Without this figure, the contribution of genetics in AIS etiology could not be compared with that of other common traits. For example, the heritability of body height was estimated to be between 0.89 to 0.93 [14], which indicated that a significant proportion of variation in body height was accounted for by genetic factors. Similarly, genetic liability to disease could be evaluated in terms of heritability.

Tang et al. examined the sibling recurrent risk and heritability of AIS in first-degree relatives of 415 Chinese patients who were first diagnosed by a community screening program [15]. Out of the total 531 sibs, 94 sibs had scoliosis (17.7%). The prevalence of AIS among male and female sibs were 11.5% (95% CI: 7.5–15.5%) and 23.0% (95% CI: 18.1–27.9%), respectively. These recurrent risks were significantly higher than the risk in the general population (*p* < 0.0001) supporting a strong familial tendency of AIS. The average sibling recurrent risk ratio was 13-fold higher than that of the general population. Overall, heritability for AIS was estimated to be 87.5%, which was comparable to that of body height. 

These results support the prevailing impression of a strong genetic liability for development of AIS [13]. Due to the polygenic nature of complex traits, inheriting genetic risk alleles does not definitely result in the disease. It just represents one additional risk factor among many others. The final phenotype outcome might represent an integration of both genetic and environmental (including lifestyle) factors. This was the first report of the standard genetic aggregation parameters for sibling recurrent risk and heritability of AIS in the Chinese population. The findings confirm that AIS has a surprisingly strong genetic predisposition which is comparable to that of other common diseases or complex traits. Such a high level of heritability was also later confirmed in Caucasian AIS patients who were recruited among those with more severe disease (higher Cobb angle or received surgery) [16,17]. The results of sibling recurrent risk were also replicated and validated in other populations as well [18].

Therefore, a high heritability of AIS liability is (1) universal across populations and (2) applied to both mild and severe disease [16]. However, the figures for heritability may be overestimated due to study design and methodology; for example, there are shared environmental factors among siblings in addition to shared inheritance in the family. Although a high level of heritability was found by different methodology [13,15], the exact contribution of the shared environmental factors in relation to the heritability of AIS liability is difficult to determine. 

## 4. AIS Delineated into 2 Phases: Initiation and Progression

Heterogeneity both in terms of phenotype and genotype are enormous in AIS. It was widely reported that discordant profiles of spine curvature are present among siblings in a family [17,19] and even between genetically identical twins [20,21,22]. On the other hand, these studies confirm a very high concordance rate and degree of familial segregation, thus supporting the conclusion of the high heritability of AIS. At first glance, these two observations may seem contradictory, as there is a high heritability on one hand and low reproducibility of specific phenotype of spine deformity on the other.

**Figure 5:**
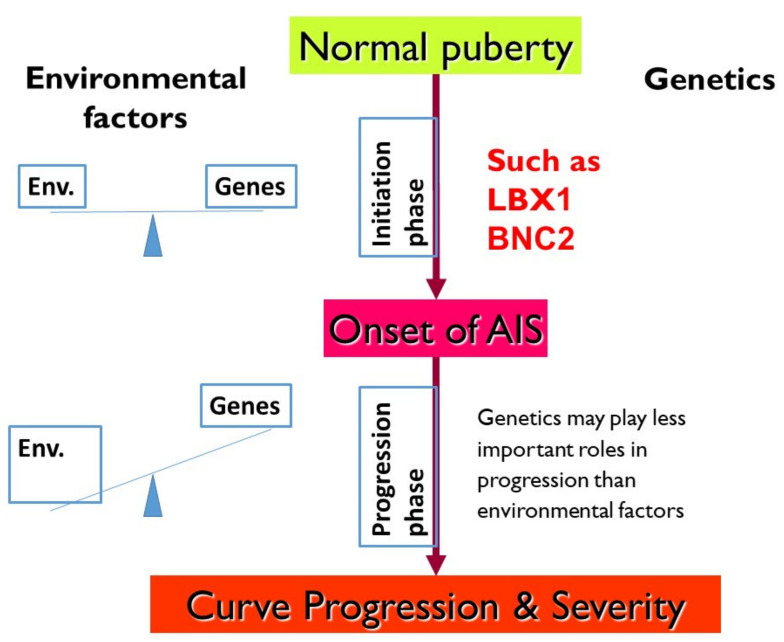
Updated 2-phase concept of AIS. With more genetic studies, more information could be filled in compared to that of first version we proposed more than a decade ago. Genes like LBX1 and BNC2 are AIS predisposition genes that contribute to initiation phase. On the other hand, environmental (Env.) factors may outweigh genetics in determining how far curve would advance.

To reconcile these observations, we proposed to understand AIS in two phases. Firstly, an initiation (onset) phase when the spine curvature starts to form, and a second progression phase, which determines the primary direction, severity, and the outcome of the curvature. With this 2-phase concept, the contradictory observations mentioned above could be reasoned if different genetic and environmental factors operate at different phases. Firstly, some genetic and environmental risk factors trigger the initiation of curvature. Then, it is followed by a separate battery of factors (i.e., genetic and/or environmental), which determine how far the curve would advance during the progression phase. In 2007, Cheng et al. elaborated this concept [4]. Figure 5 shows an updated version of the concept. Subsequently, this scheme and nomenclature were cited by other researchers in their publications [5,16] and implemented into orthopedic textbooks.

The implication of the 2-phase model is far-reaching. In the past, the traditional research approach lumped the 2 phases together and results were poorly reproducible due to risk factors of different phases that were confounding each other. Recent research design incorporates the 2-phase model and leads to ground-breaking discoveries of disease predisposition genes in initiation of AIS (to be discussed in the next section).

While genetic studies are very informative, it is important to tease out false-positive associations. That is why replication of studies and confirmation of association signals in other sample sets, or even better in other population samples, are fundamental principles of high-quality genetic studies. While predisposition genes leading to the initiation of spinal curvature were found and validated by different studies, genetic findings were poorly reproducible for prediction of the extent of curve progression (commonly represented by the final Cobb’s angle). A biotechnology startup company developed a single nucleotide polymorphism (SNP) based prognostic test to predict curve progression among AIS patients [23]. It is composed of genotype data of 53 SNPs, and the resulting data divided patients into low, medium, and high risk for curve progression. Query was raised early after the first publication as it appeared that the test was only good at the identification of those patients without curve progression [24]. Subsequently, the initial results and potential utility of the test could not be replicated in multiple secondary studies involving both Caucasian and Asian patients [25,26,27,28].

Collectively, data are suggestive that the progression phase is potentially under secondary influences including both environmental influence and genetic effects together with potential epigenetic influence. This hypothesis was further supported by the lack of correlation between curve severity and family history of scoliosis [29]. Therefore, we depicted with more detail the 2-phase model in Figure 5, which highlights the differential influence of genetic and environmental factors in each phase. While genetic factors predominantly determine the liability during the initiation of the curve, environmental/together with (epi) genetic factors may govern how far the curve will progress. Many factors are involved in curve progression, such as basic demographics (age of onset, sex), growth velocity, bone mineral density [30], mobility, and morphology of the scoliosis spine on imaging [31,32]. The effect on curve progression due to anthropometric, environmental, and lifestyle factors are reviewed in these publications [33,34,35].

## 5. Genetic Association Study in AIS and GWAS

In view of the complex trait nature of AIS, genetic association studies would be the most appropriate methodology in finding the susceptibility genes. Linkage analysis typically used a few families [36] and few of them resulted in identification of a specific gene involved (for example, [37]). In general, conventional linkage analyses were not particularly successful in finding causative genes for AIS. Linkage studies with even a large number of families did not arrive at convincing predisposition genes by using the standard linkage analysis [6,7]. 

The issue of genetic heterogeneity in AIS is also evident in linkage study results, as no single locus found by linkage analysis was replicated by another research center. This lack of overlap in the reported linkage loci may be a result of several reasons. Firstly, they might be false-positive linkage loci. Secondly, it is also plausible that every family has their own private locus, which implies that many scoliosis causative genes are present in the genome. Furthermore, a genealogical study of IS patients from the Intermountain West [3] also suggested multiple different genes might be involved in the predisposition of Idiopathic Scoliosis in different families. Currently, the exact reason for this lack of replication of linkage results is not known and both possibilities are equally valid.

In the 2007 annual Cotrel foundation research meeting, the genetic association study methodology was proposed. The complex trait paper was written and published before the era of genome-wide association study. The objectives of the earlier research supported by the Cotrel Foundation were to perform genetic association studies and to fine map a previously linkage-supported locus and other candidate genes. On the other hand, genetic association analysis of a dichotomous (discrete) trait may be more suitable to identify genetic etiology of the multifactorial complex trait that could account for a great proportion of AIS patients [4].

**Figure 6:**
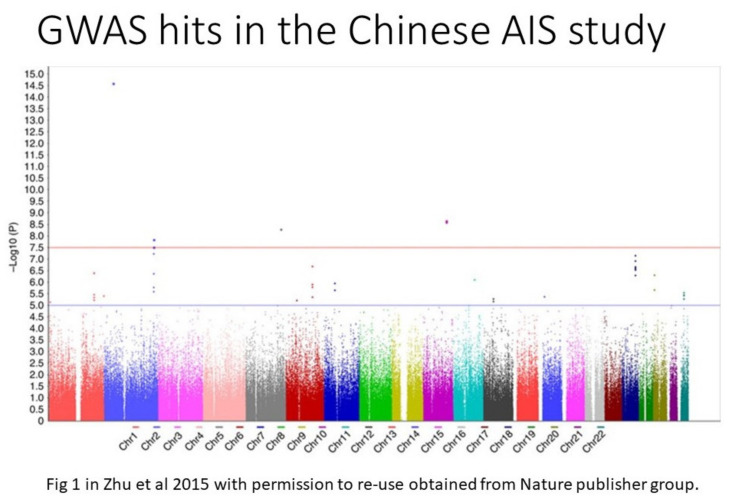
An example GWAS results in AIS showing putative predisposition SNPs and their statistical significance in a Manhattan plot from Chinese AIS GWAS study [42].

While genome-wide association study (GWAS) would provide an unbiased representation of association signals across the whole genome, large funding support was needed. Immediately after the first GWAS WTCCC paper [38], the Cotrel research group and others gathered in Paris to create a proposal to perform a GWAS in AIS. Although the GWAS budget exceeded that of the regular grant funding mechanism, the Cotrel Foundation arranged a special meeting in 2008, invited leading scientists, including Prof. Francois Gros and Prof. Stuart Edelstein to discuss issues related to genetics of AIS. Subsequently, a research team in Riken, led by Prof. Ikegawa, reported the discovery of LBX1 gene in a GWAS of Japanese AIS patients in 2011 [39]. Although this gene was not detected by another GWAS with only 419 AIS families [40], the LBX1 gene turned out to be the first predisposition gene that was then replicated in multiple subsequent studies. Londono et al. also replicated the finding as part of the International Consortium for Scoliosis Genetics [41]. Similar to most other predisposition alleles in other complex traits, the SNPs carried a small increase in the risk of AIS (Odds ratio~1.5 fold for the high risk allele of rs11190870), and thus, accounted for a small fraction of the heritability of AIS. Additional genetic predisposition loci are yet to be found. In 2015, Zhu et al. reported the first GWAS in Chinese AIS patients [42,43] (as illustrated in Figure 6). These and other latest GWAS confirmed the complex trait nature of AIS and collectively they formed the category of low penetrance genes leading to the initiation (onset) of AIS. 

## 6. LBX1 as the First Confirmed AIS Genetic Predisposition Locus

The research field understands that false-positive findings are common in candidate gene associations due to spurious associations. GWAS is also susceptible to spurious association as it performs so many statistical tests in one study. Although stringent Bonferroni correction is applied to define the cutoff *p*-values, it does not guarantee all GWAS hits are true positives. Replication provides the ultimate validation of any association [44,45]. LBX1 is the most highly replicated AIS predisposition locus to date [39,41]. However, the biological role of LBX1 in AIS is largely unknown. LBX1 is a member of a large family of homeobox transcription factors and regulates upper limb muscle precursor cell migration during embryo mesoderm development in chicken and mice [46,47,48]. LBX1 has a long evolutionary history in most chordates and is required for the formation of the neuromuscular unit of hypaxial or upper limb musculature [48]. LBX1 also regulates the differentiation and maintenance of satellite cells in postnatal mice muscle [49]. In contrast, the role of LBX1 on bone is less obvious. 

Genetic associations were detected near to but outside the protein-coding sequence of LBX1 gene (for example, rs11190870, rs625039, and rs11598564 are intergenic SNPs associated with AIS), together with rs678741 in a nearby antisense transcript called *LBX1-AS1*. The predisposition allele of rs11190870 was the major (prevalent or common in the healthy population) allele in various populations with an allele frequency ranging from 0.49 to 0.58) [41,50]. It is unknown why such a prevalent allele would predispose to a prevalent disease, as the challenge of natural selection would be expected to select out defective (disease predisposition) alleles or for those alleles to become less frequent.

## 7. BNC2 Is Another Replicated AIS Locus Related to Muscle Development

Rs3904778 located in an intron of BNC2 was first associated with AIS in a GWAS of Japanese patients [51]. The high-risk haplotype was suggested to bind to a muscular cell transcriptional factor, YY1. The results were further replicated in Chinese patients and Caucasian patients [51,52,53].

Both LBX1 and BNC2 are functionally related to early muscle development. These findings provide potential new insight into the initiation (onset) of scoliosis. AIS was traditionally viewed as a bone disease involving the various levels of vertebrae and their growth. With these highly replicated genetic predisposition loci identified, which do not have an obvious function in bone, the traditional understanding of AIS as a primary bone disease needs to be reconsidered. Instead, a primary functional alteration of muscle with subsequent interplay between muscle and bone tissues may be occurring during the initiation of scoliosis.

## 8. Extracellular Matrix and Fibers

Patients with Marfan syndrome and Ehlers–Danlos syndrome often develop scoliosis among other inherited types of connective tissue diseases. They are autosomal dominant diseases with mutations in fibrillin (FBN1) or one of the collagen genes [54,55]. Rare mutations in FBN1 and FBN2 were associated with scoliosis, even though these scoliosis patients do not have typical features consistent with the syndromic condition. In addition, it was the load of mutations among these genes rather than any single gene that determined the predisposition to AIS [56,57].

## 9. Fish Studies Confirmed Scoliosis as a Complex Trait

A major hurdle in the study of AIS is the development of a reliable animal model that is representative of the disease phenotype. Previous attempts of inducing scoliosis with surgical intervention, such as using pinealectomy chicks and bipedal mice, were largely unsatisfactory. A spontaneous scoliosis animal model with spinal curvature developing during a rapid growth phase (reminiscence of the pubertal growth) was long sought. Zygotic ptk7 (Protein tyrosine kinase-7) mutant zebrafish provide important clues to the etiology of scoliosis and represent a breakthrough in animal model research of AIS [58,59]. Ptk7 and planar cell polarity (PCP) signaling are normal during the embryonic period as supported by maternal ptk7 deposited in the eggs which facilitate normal morphogenesis. Only after physiological degradation of maternally derived gene products will a deficiency of ptk7 occur in the larval stage of the transgenic fish [60]. This indicates that a single defect during the postnatal growth phase can cause scoliosis. Being an essential pathway, PCP signaling is involved in various pathologies related to embryonic development, such as neural tube defects [61,62,63]. While the ptk7 fish model faithfully demonstrated the vertebral curve phenotype during its rapid growth phase, unfortunately, the mutant fish also developed hydrocephalus, which was attributed to defects in ependymal cell cilia function [50]. Despite this, the results have far-reaching implications. Unlike those induced scoliosis in chicken and mice models, it was one of the few models that best resembled the situation in human disease. Another one was recessive kif6 mutant in zebrafish [64]. The ptk7 mutant has an additional advantage that the onset of spinal curvature is later in the life cycle and coincides with the rapid growing juvenile stage.

In zebrafish, due to the small CSF space, cilia may be essential for a normal CSF flow. On the other hand, Chu et al. measured the CSF flow in patients with neural axis anomalies but failed to demonstrate any observable impairment [65]. A study by Zhang et al provided the mechanism linking abnormal CSF flow and scoliosis [66]. They found that cilia action was essential to maintain CSF flow in the zebrafish embryos as well as the transport of adrenergic signals for neuromuscular development. Both CSF flow disruption and urotensin receptor mutations induced scoliosis in zebrafish [67,68]. The results suggest that neuromuscular development of the dorsal somite is indeed the common and critical pathway leading to scoliosis and that the cilia abnormality was potentially a trigger for the spinal curvature instead of being an essential mechanism. The findings in zebrafish reinforce the concept that initiation of AIS may be due to a neuromuscular problem rather than being a primary bone disease. Similar scoliosis-related phenotypes were also induced in other zebrafish mutants (for example, stat3 and myh3) although the mechanisms may be different [69,70].

With these scoliosis of spontaneous-onset fish models, various treatment strategies can now be examined. Traditional scoliosis treatments target primarily mechanical correction by using bracing or surgery. Few attempts of medical treatment other than melatonin were tried. Melatonin treatment was based on the pinealectomy induced scoliosis model in chicken, however, the efficiency in patients was limited [71]. The zygotic mutant zebrafish established that the onset of scoliosis in the zebrafish model could be ameliorated by drug treatment alone. Treatment with aspirin or N-acetylcysteine lowered the prevalence of scoliosis in ptk7 mutant zebrafish [72]. These treatments either delayed the onset of scoliosis or reduced the prevalence of scoliosis. For example, the prevalence of scoliosis reduced from 81% to 17% after treatment with N-acetylcysteine [72]. These early zebrafish findings provide new hope for potential medical intervention in human AIS. If such nonsurgical interventions can be potentially effective, they will represent a major milestone in scoliosis treatment.

A key difference between single-gene disease and the polygenic complex trait is that the role played by environmental factors should have a prominent contribution in the latter. Cheng et al and other researchers showed that systemic low bone mineral density (BMD) was a significant and independent prognostic factor for curve progression. Furthermore, over 30% of AIS had low BMD as compared to 16% in that of the general population [5]. Low BMD results from gene–environmental interaction and can be considered as an environmental factor contributing to liability to AIS. Osteopenia represents a risk factor that could be modified, and it remains to be seen if such interventions could have beneficial therapeutic effects on scoliosis.

## 10. Discussion and Looking Forward

### 10.1. What Genetic Studies Inform about the Etiology of Scoliosis?

This is a question frequently raised by colleagues: how can genetic studies benefit patients? It is an excellent question, and it deserves an answer. We hope that after reading this review, many colleagues will appreciate that the knowledge and understanding of AIS were much enhanced. More than 20 years ago, Dr. Cotrel initiated the visions as shown in Figure 1 in which he figured out 10 potential categories of pathogenesis that were equally likely to cause AIS. Now, we have arrived at strong data supporting genetic loci in muscle developmental genes and extracellular matrix as potential predisposition genes.

The next question is whether such understanding could be obtained without utilizing genetic studies. We believe that the advances in the past decade are largely related to genetic research. Genetic studies have a unique advantage that all genetic alleles, mutation, or genetic risk factors must be the primary event in any causative analysis. Identifying a causative chain of events, and determining which are the primary and secondary adaptive events in pathogenesis is always difficult. Furthermore, it was a century-long argument about which tissue (representing the 10 potential categories of pathogenesis in Figure 1) holds the primary defect in AIS (be it bone, cartilage, ligament, spinal cord, muscle, nerve, intervertebral disc, etc.). In addition, other research approaches could only collect samples from patients requiring surgery who, by default, must have an advanced curve and tissues are obtained late during the disease process. The pathology found in these settings is virtually impossible to differentiate between the primary causative change or secondary adaptive response. On the other hand, alleles and mutations in a gene used for a particular function are fixed at the time of conception. Therefore, it predates the onset of any disease so that mutations and alleles must represent a primary event and pinpoint the functional pathway of pathogenesis. When working with genetic association or linkage results, the concern regarding the differentiation between primary cause or secondary adaptation is largely diminished. Our task is merely (1) to confirm that the genetic findings are genuine and not false positive, and (2) to make biological sense from the findings to understand the underlying functional defects.

LBX1 and BNC2 are primarily related to early muscle development. Fibrillin and collagen genes form the extracellular matrix and connective tissue. They stand out among others in GWAS and mutation analysis of AIS and provided strong support that pathogenesis of a proportion of AIS patients is related to soft tissue abnormalities. Complex traits are characterized by patient heterogeneity meaning that patients may have different etiologies. Given the understanding of complexity in AIS, soft tissue anomalies could be an initiation event in a significant proportion of AIS patients, in particular since LBX1 risk alleles are so prevalent in the population. We think that these findings may help to settle the century-long argument of the tissue of primary defect in AIS and provide an answer to Dr. Cotrel’s questions about the 10 categories of etiologies. With this new concept, new innovative approaches to treatment and management can be envisioned. It is hoped that the results of research in this field will help a proportion of patients whose scoliosis are due to soft tissue pathogenesis to reduce the morbidity of AIS in the future.

### 10.2. What Should Be the Future Research Direction?

The identification of soft tissue as a potential primary defect leading to the initiation of spinal curvature will be an important direction for future research. It suggests that the trajectory or disease path after AIS onset is amenable to modification. Two new ideas for future research direction are obvious with these findings: (1) early curve control/correction, and (2) screening for early curve.

**Figure 7:**
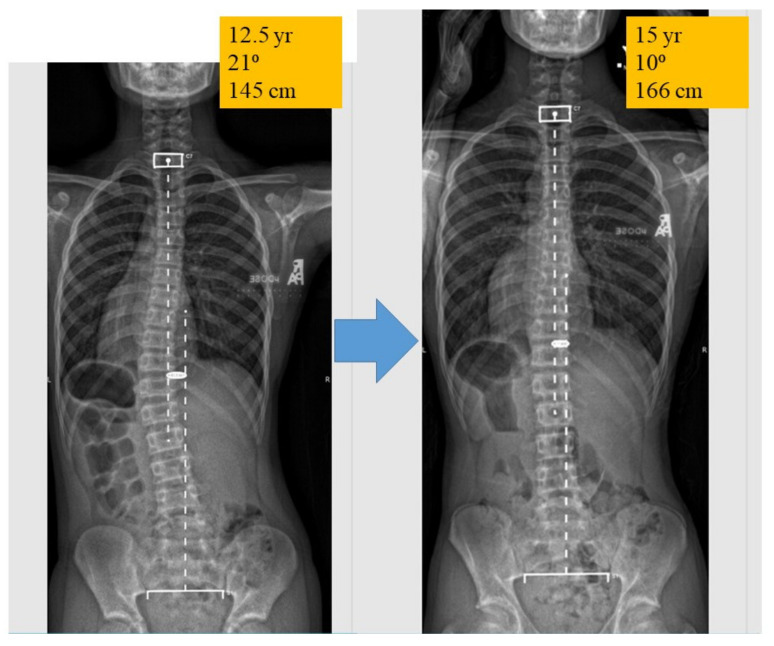
Spine X-rays of a male patient who was first diagnosed at 12.5 years old with a 21-degree curve. Environmental and lifestyle modifications were implemented like upper limb exercise, posture awareness, and change of seating furniture. His curve reverted to 10 degrees at the age of 15 years old and the follow-up x-ray was taken after he had passed through his peak growth spurt. Bracing was not prescribed in this case. This case demonstrates that soft tissue remodeling is feasible in some selected patients.

Remodeling of soft tissue is more feasible than remodeling hard tissue. For example, soft tissues can be remodeled by various means, e.g., exercise, stretching, bracing, etc. Inspired by the soft tissue remodeling idea, Figure 7 shows the follow-up X-ray of a male AIS patient whose curve was controlled with lifestyle and environment modifications alone without bracing.

Bracing provides regional control of the spine curvature and was used for a long time. However, there is no biological ground to determine when bracing should be started. The implication that being a soft tissue disease in some AIS patients from the outset would suggest that we need to start bracing early in the course of disease before secondary adaptive bone changes occur which perpetuate a vicious cycle of maladaptation.

For an early intervention to become available and effective, patients need to be identified early. As a matter of fact, early diagnosis is also required to evaluate the effectiveness of any early intervention. Hong Kong is among the few regions in the World that practice universal scoliosis screening of school children. Screening enables detection of early patients with milder curves, whereas hospital cases are usually biased toward severe cases so that a comprehensive, full-spectrum understanding of the epidemiology of AIS is possible. In addition, patients with mild curves could be studied so that risk factors for curve progression can be analyzed. The recent advance in low-dose radiation imaging instruments allows accurate and close monitoring of curve progression which was not possible before. These new technologies will very likely bring us new understandings of the biomechanics of early stage of disease when targeted intervention could be possible. All such new concepts, of course, need further evidence and clinical trial to support.

In conclusion, genetic studies of AIS yielded breakthroughs in the understanding of its etiology. Genetic predisposition in genes related to muscle development and extracellular matrix components indicates that initiation of AIS in some individuals is potentially due to soft tissue pathology. The new insights clarify the dated arguments in pathogenesis and provide new directions for future research and potential treatment of the disease.

## Data Availability

Not applicable for this review.
